# Optimization of the Shape of Hooked-End Steel Fiber Based on Pulling Out and Reinforcing Cementitious Composites

**DOI:** 10.3390/ma17010047

**Published:** 2023-12-21

**Authors:** Xiaowei Wang, Bo Xu, Kuiliang Luan, Ru Mu, Jiao Chen

**Affiliations:** School of Civil and Transportation Engineering, Hebei University of Technology, Tianjin 300401, China; xubory@163.com (B.X.); l753816472@163.com (K.L.); jiao_chen0712@163.com (J.C.)

**Keywords:** fiber concrete, steel fibers, pullout strength, residual pullout resistance, L/D ratio

## Abstract

Efficient steel fiber—reinforced cementitious composites (SFRCC) should improve not only the ultimate tensile strength but also the residual tensile strength (post-cracking tensile strength) of the SFRCC matrix. The degradation of the post-cracking tensile strength of SFRCC depends on the pullout properties of the steel fibers from the cementitious matrix. When the straight steel fiber was pulled out from the matrix, the pullout resistance was affected by the actions of bonding and friction. After debonding, the load was transferred only by friction, and the pullout resistance decreased rapidly, resulting in a weak reinforcing effect. In addition, changing the fiber shape can effectively slow down the decreasing rate of the pullout resistance of the steel fibers, thus improving their reinforcing effects. In this study, the shape of the steel fibers was optimized to slow down the decrease in the pullout resistance of the steel fibers from the cementitious matrix, thus achieving better tensile properties of SFRCC. First, a calculation model for the steel fiber pullout resistance was established. On this basis, a method to optimize the shape of the steel fibers was proposed. Finally, the pulling out behavior of steel fibers with different end hook shapes was tested, and the influence of the steel fiber shape on the decreasing rate of the residual pullout resistance was analyzed. The results showed that the optimized hooked-end steel fibers had better pullout resistance than ordinary hooked end steel fibers of the same diameter, tensile strength, and matrix.

## 1. Introduction

The incorporation of steel fibers into the cementitious matrices can overcome their disadvantages, such as low tensile strength and high brittleness [1,2]. When cracks occur in steel fiber—reinforced cementitious composite (SFRCC) members, the steel fibers at the cracks provide a bridging effect, enabling the members to withstand the load and avoiding brittle damage [3,4,5]. For SFRCC, a higher tensile capacity (strength) and a slower decrease in tensile capacity (residual tensile strength) after cracking indicate a better performance [6,7]. Unlike the brittle failure of plain cement—based composites, the residual tensile strength is an essential difference between steel fiber—reinforced cement—based composites and ordinary cement—based composites. Therefore, the residual tensile strength is the key performance index of fiber—reinforced cement—based composites.

The tensile properties of SFRCC depend on the pulling out behavior of the steel fibers as they are pulled out of the matrix [8,9]. When a single straight steel fiber is pulled out from cementitious composites, the pullout resistance is mainly derived from bonding and friction effects [10]. After debonding, the load was transferred only through friction. If the fiber length is small, the pullout resistance after debonding decreases rapidly, with limited reinforcing effects on the matrix. Increasing the fiber length can improve the maximum pull out resistance of the steel fibers and slow the rate of decrease in the residual pull-out resistance. However, increasing the fiber length was not as effective as changing the shape of the fiber. Changing the shape of steel fibers can produce mechanical anchorage during the pullout process, thus effectively improving the pulling out behavior [11,12]. In addition to improving the pullout resistance of steel fibers, a reasonable end—hook shape can also prevent fiber breakage due to excessive anchoring force and avoid steel fiber defects caused by end—hook processing. Adjusting the shape of the steel fibers to slow down the decrease in the pullout resistance of the steel fibers from the cementitious matrix can lead to better tensile properties of SFRCC, which is of great significance for practical engineering.

## 2. Calculation Model of Steel Fiber Pullout Resistance

**Figure 1:**
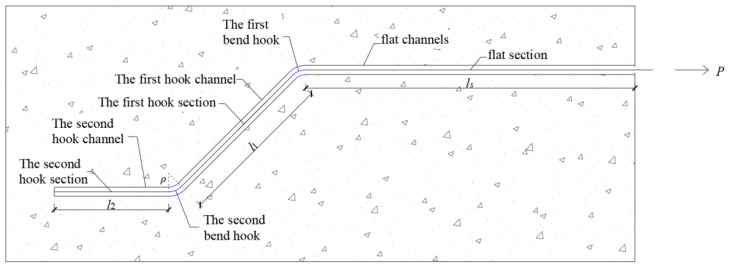
The schematic diagram of the hooked-end steel fiber embedded in the matrix.

When a common straight steel fiber is pulled out of a cementitious matrix, the pullout resistance is affected by bonding and friction [10]. After debonding, the steel fiber is only affected by friction, with a rapid decrease in the pullout resistance [10]. By adding a hooked end, the fibers are mechanically anchored during the pullout process, thus improving the pullout resistance of the steel fibers from the cementitious matrix and slowing the decrease in the pullout resistance [11]. The hooked end steel fibers embedded in the matrix are shown in Figure 1. As the hooked end steel fibers begin to be pulled out of the matrix, they steel fibers are in a fully bonded state to the matrix [13]. When the pullout resistance of the steel fiber reached the maximum bonding force, the steel fiber started to debond from the side of the loading end [13,14]. When debonding occurred at the 1st hook, the mechanical anchoring effect is initiated. As debonding continued, mechanical anchoring was exerted at the 2nd hook until the fibers were completely debonded from the matrix. After complete debonding, the pullout resistance of the hooked end steel fiber consists of the mechanical anchoring force of the end hook and the friction force between the fiber and matrix in the flat straight channel, with the maximum pullout resistance in this state [15]. Subsequently, the displacement of the loading end continued to increase, the mechanical anchorage force of the end hook decreased, and the pullout force decreased. The pullout resistance at this point was regarded as the residual pullout resistance. A pullout resistance model of the steel fibers was established by analyzing the pullout resistance of the steel fibers in each section.

### 2.1. Mechanical Anchoring Force of Hooked-End

**Figure 2:**
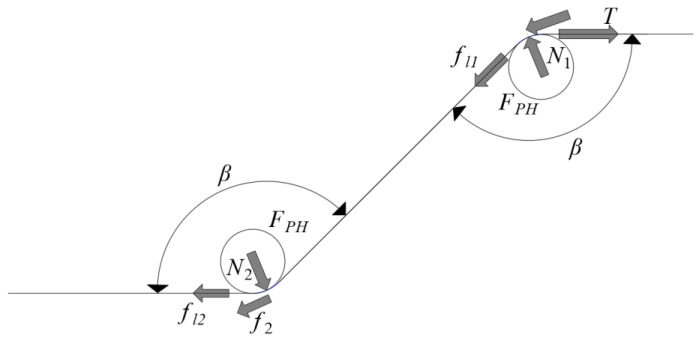
Force analysis at the end hook of hooked-end steel fiber [16].

In the pullout process, the end hook is gradually straightened, and the force analysis when the end hook exerts a mechanical anchoring force is shown in Figure 2 [16,17].

According to the equilibrium relationship between the fiber pullout resistance and pullout force, the pullout force *T* on the end hook is equal to the mechanical anchoring force *T_H_* (pullout resistance, which is the total resistance generated by the end hook). The mechanical anchoring force of the end hook is the sum of the *F_PH_* (when the hooked end steel fiber is pulled out, cold bending deformation occurs at the hook, that is, plastic deformation, and *F_PH_* is the force that causes plastic deformation at the hook. It belongs to the pullout resistance) and friction force (pullout resistance) required to produce plastic deformation at the hook. The end-hook mechanical anchoring force *T_H_* can be calculated by Equation (1).

(1) $$T_{H}=T=2F_{PH}+f_{l1}+f_{l2}+f_{1}+f_{2}=\frac{2F_{PH}+{\left(1+\mu cos\frac{\beta }{2}\right)}^{2}f_{l2}+\left(1-\mu^{2}{cos}^{2}\frac{\beta }{2}\right)f_{l1}}{{\left(1-\mu cos\frac{\beta }{2}\right)}^{2}}$$

where *T_H_* is the mechanical anchoring force of the end hook; *T* is the pullout force on the end hook; *F_PH_* is the force required to produce plastic deformation at the hook; *f_l1_* and *f_l_*_2_ are the friction forces on the fibers in the 1st and 2nd hook channels, respectively, $f_{l2}={\tau_{s}\pi d}_{f}\left(l_{1}\;-\;∆\right)$; *τ_s_* is the interfacial shear stress between the steel fibers and the matrix; *d_f_* is the diameter of the steel fibers; *l_1_* is the length of the 1st hook channel; Δ is the loading end displacement; *f_1_* and *f_2_* are the friction forces generated at the 1st and 2nd hooked ends, respectively; *β* is the angle of the steel fiber hooked-end; *μ* is the friction coefficient between the steel fibers and the matrix; $\mu =0.25+\left(0.95\sqrt{f_{ck}}-3.8\right)/21.28$ [16], *f_ck_* is the compressive strength of the matrix.

Based on numerous test results and theoretical analyses, Chanvilard et al. [18] obtained the relationship between the force *F_PH_* required to produce plastic deformation at the hook and the bending moment *M_PH_* applied to the circular section of the hook, as expressed by Equation (2).

(2) $$F_{PH}=\left(1.18-\frac{M_{PH}}{M_{P}}\right)F_{P}$$

where *M_PH_* is the bending moment applied to the circular cross section when plastic deformation occurs at the hook, which can be calculated using Equation (3) [19]; *M_P_* and *F_P,_* indicate the bending moment and tension force applied to the circular cross section at the hook of the steel fiber under a complete plastic state, respectively, $M_{P}=4f_{y}{r_{f}}^{3}/3$ and $F_{P}=\pi f_{y}{d_{f}}^{2}/4$; *f_y_* is the yield strength of the steel fiber; and $r_{f}$ is the radius of the steel fiber.

(3) $$M_{PH}=4f_{y}{r_{f}}^{3}\left[\frac{1}{sin\theta }\left(\frac{\theta }{8}-\frac{sin(4\theta )}{32}\right)+\frac{{cos}^{3}(\theta )}{3}\right]$$

where $\theta =arcsin(\rho f_{y}/E_{s}r_{f})$; *ρ* indicates the radius of curvature of the hook, *E_s_* represents the elastic modulus of the steel fiber.

**Figure 3:**
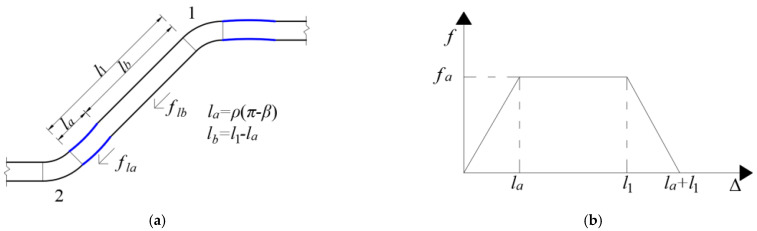
Force analysis of fiber in the first hook channel. (**a**) The force schematic diagram of the fiber in the first hook channel; (**b**) The relationship between the additional friction and the displacement of the loading end.

Friction *f_l_*_1_ applied to the fibers in the 1st hook channel consisted of the additional friction generated by the unstraightened part at the 2nd hook and the friction applied to the straight part of the 1st hook, as blue the portion shown in Figure 3a. The variation in the additional friction with the displacement of the loading end is shown in Figure 3b (*l_a_* is the length of the bending section at the hook) [20]. When the 2nd hook completely slips into the 1st hook channel, the friction *f_l_*_1_ applied to the fibers in the 1st hook channel can be calculated using Equation (4).

(4) $$f_{l1}=f_{la}+f_{lb}=\frac{1}{2}\left[-0.75\left(f_{c}+f_{y}\right)\frac{{d_{f}}^{3}}{l_{e}}+0.8205{d_{f}}^{2}\sqrt{f_{c}f_{y}}\right]+\tau_{s}\pi d_{f}\left[l_{1}-\rho \left(\pi -\beta \right)\right]$$

where *f_la_* is the additional friction generated by the unstraightened part of the hook; *f_lb_* is the friction force on the straight part of the hook; *f_c_* is the axial compressive strength of the matrix; *l_e_* is the total length of the steel fiber embedment, $l_{e}=l_{1}+l_{2}+2\rho \left(\pi -\beta \right)+l_{s}$.

When the end part of the steel fiber slips to the 1st hook channel, the *F_PH_* and friction *f_l_*_2_ and *f*_2_ to produce plastic deformation at the 2nd hook disappear. At this time, the end hook mechanical anchoring force *T*_2,1_ is the sum of *F_PH_* and friction *f_l_*_1_ and *f*_1_ required to produce plastic deformation at the 1st hook, as expressed by Equation (5).

(5) $$T_{2,1}=F_{PH}+f_{l1}+f_{1}=\frac{\left(1+\mu cos\frac{\beta }{2}\right)f_{l1}+F_{PH}}{1-\mu cos\frac{\beta }{2}}$$

where *T*_2,1_ is the mechanical anchoring force of the end hook when the end of the steel fiber slips into the 1st hook channel.

### 2.2. Friction between the Fiber and Matrix in a Flat Channel

Additional friction also occurs in the unstraightened part of the hook after the hooked-end steel fiber slips into the flat channel of the 1st hook. Therefore, the force analysis of the fibers within the straightened channel is similar to that of the fibers within the 1st hook channel (Figure 3). The friction on the fibers in the flat channel consists of the additional friction generated by the unstraightened part at the hook and the friction applied to the flat section within the matrix, as expressed in Equation (6).

(6) $$f_{ls}=f_{la}+f_{lc}=\frac{1}{2}\left[-0.75(f_{c}+f_{y})\frac{{d_{f}}^{3}}{l_{e}}+0.8205{d_{f}}^{2}\sqrt{f_{c}f_{y}}\right]+\pi d_{f}\tau_{s}\left(l_{s}-∆\right)$$

where *f_ls_* is the fiber friction in the flat channel, *f_la_* is the additional friction generated by the unstraightened part at the hook, *f_lc_* is the friction applied to the flat section within the matrix, *l_s_* is the length of the flat section.

### 2.3. Maximum and Residual Pullout Resistance

Both the maximum and residual pullout resistances of the hooked end steel fibers consisted of the mechanical anchoring force of the hooked end and the friction of the fibers in the flat channel. When the unstraightened section at the hook reaches its maximum friction, both the mechanical anchoring force of the end hook and the fiber friction within the flat channel achieve their peak levels. At this point, the pullout resistance reaches its maximum value. The residual pullout resistance was significantly reduced due to the straightening of the hook. Therefore, the pullout resistance of the freshly straightened hook was selected to analyze the residual pullout resistance. The maximum pullout resistance of the steel fiber and the residual pullout resistance of the freshly straightened hook can be expressed by Equation (7).

(7) $$P\left(∆\right)=\left\{\begin{array}{l} P_{max}=T_{H}+f_{ls}\;\;\;\;\;\;\;\;\;\;\;\;\;\;∆=\rho \left(\pi -\beta \right)\\ P_{R}=T_{2,1}+f_{ls}\;\;\;\;\;\;\;\;\;\;\;\;\;\;\;\;∆=l_{2}+\rho \left(\pi -\beta \right) \end{array}\right.$$

where *P*_max_ is the maximum pullout resistance of the hooked end steel fiber, and *P_R_* is the residual pullout force of the freshly straightened hook.

Similarly, the maximum and residual pullout resistances corresponding to the steel fibers with different hook numbers were obtained.

## 3. Optimization of Hooked-End Steel Fiber Shape

Adjustment (optimization) of the steel fiber shape can increase the maximum and residual pullout resistance of the steel fibers during pullout from cementitious composites. As a result, the reinforcing effect of steel fibers can be enhanced, thereby improving the performance of SFRCC. For the commonly used hooked end steel fibers, the maximum pullout resistance is first increased by adjusting the hook angle. The decrease in pullout resistance with fiber slip under tension is slowed, indicating that the pullout resistance was well maintained. If the adjustment of the hook angle cannot achieve favorable results, the number of hooks must be increased before adjusting the hook angle.

As the steel fiber residual pullout resistance decreases more slowly, the ratio of the residual pullout resistance to the maximum pullout resistance at the same pullout displacement increased. The residual pullout resistance of the steel fibers increases with decreasing hook angle of the hooked-end steel fiber and increasing number of hooks. Therefore, to reduce the decrease in the residual pullout resistance of steel fibers, the ratio of the residual pullout resistance to the maximum pullout resistance is maximized by adjusting the hook angle and number of hooks.

If the parameters of the steel fiber are known, the appropriate hook angle is determined using the dichotomy algorithm. In the calculation using the dichotomy algorithm, the maximum hook angle gradually decreased, the minimum hook angle gradually increased, and the difference between the decrease rate of the residual pullout resistance of the steel fiber corresponding to the median value of the maximum hook angle and the minimum hook angle and the decrease rate of the residual pullout resistance of the steel fiber corresponding to the median value in the last cycle calculation gradually decreased. If the ratio of the residual pullout resistance to the maximum pull-out force changes by less than 1% when adjusting the hook angle, the hook angle can be considered as the optimal value.

**Figure 4:**
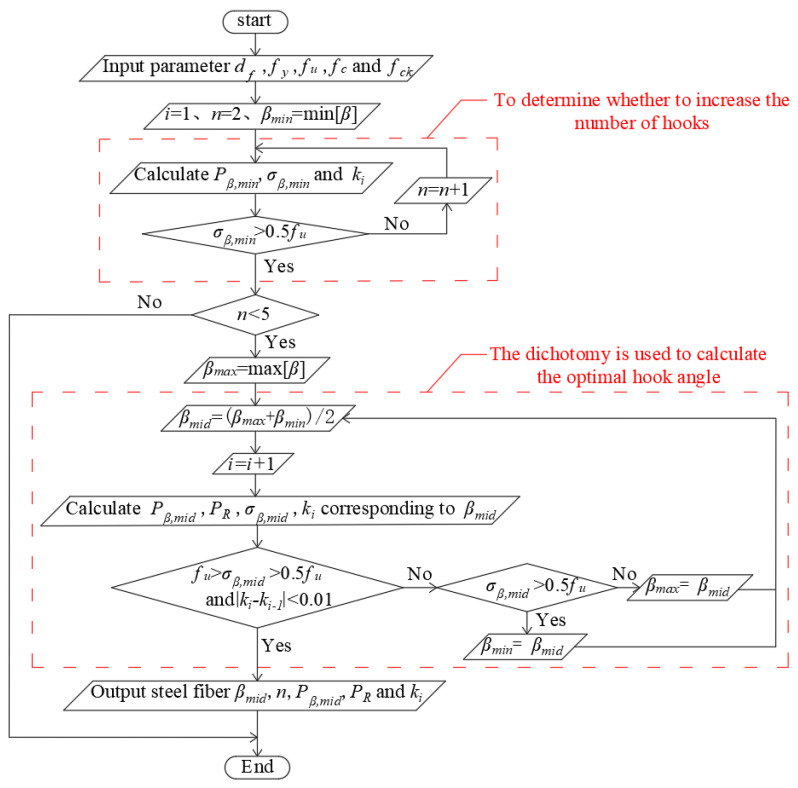
The flow chart of optimization.

The specific method is shown in Figure 4. In the figure, n is the number of hooks of the hooked end steel fiber, *β*_max_, *β*_min,_ and *β*_mid_ are the maximum, minimum, and middle angles in the calculation of the dichotomy, respectively. *P_β_*_,mid_ is the maximum pullout resistance of the steel fiber corresponding to *β*_mid_. *k_i_* is the rate of decrease in the residual pullout resistance, which is the ratio of the residual pullout resistance *P_R_* to the maximum pullout resistance *P_β_*_,mid_. σ*_β,_*_mid_ is the tensile stress corresponding to the maximum pullout resistance *P_β_*_,mid_, which is the ratio of the maximum pullout resistance to the area of the steel fiber circular section. *P_R_* is the residual pullout resistance of the steel fiber corresponding to *β*_mid_.

**Figure 5:**
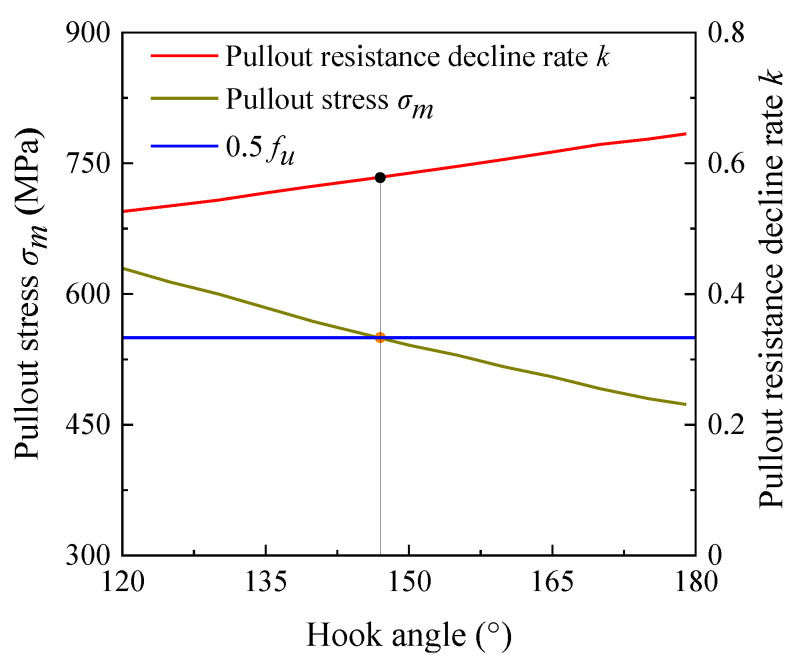
The relationship between hook angle and pullout stress and pullout resistance decline rate.

**Figure 6:**
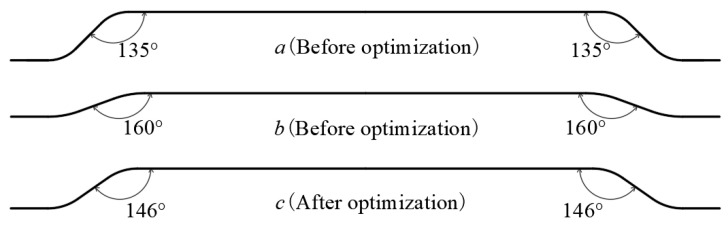
The shape of steel fibers before and after optimization.

Based on the above method, the pull out behavior of steel fibers with a diameter of 0.75 mm and a tensile strength of 1100 MPa was optimized during the pullout of steel fibers from the matrix of cementitious composites with a strength class of C40. A larger hook angle of the hooked-end steel fiber indicates a smaller pullout stress *σ_m_*, leading to a larger ratio of the residual pullout resistance to the maximum pullout resistance (i.e., a slower decrease in the residual pullout resistance, Figure 5). The pulling out behavior of the steel fibers before and after optimization is shown in Table 1, and the shape of the steel fibers is shown in Figure 6.

**Figure 7:**
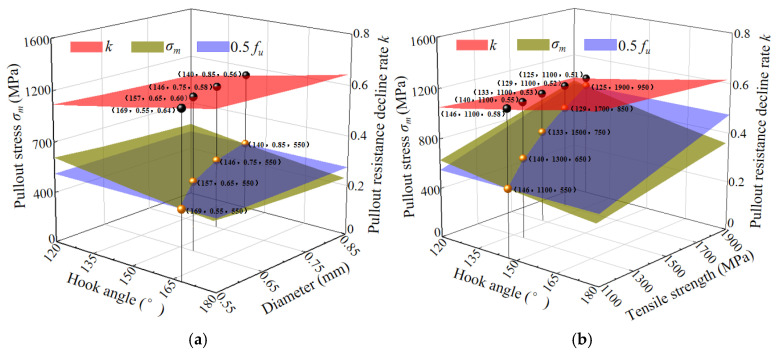
The hook angle, diameter, and tensile strength with pullout stress and the decreasing rate of pullout resistance. (**a**) The relationship of the hook angle and diameter with pullout stress and the decreasing rate of pullout resistance; (**b**) The relationship of the hook angle and tensile strength with pullout stress and the decreasing rate of pullout resistance.

Figure 7 shows the relationship between the hook angle, diameter, and tensile strength with the pullout stress and the decreasing rate of the pullout resistance.

It can be seen that a larger bending angle of the hooked-end steel fiber leads to a smaller pullout stress *σ_m_*, slowing down the decrease of the residual pullout resistance. When the pullout stress is greater than 0.5 *f*_u_, the hook angle at the intersection of the pullout stress surface and 0.5 *f*_u_ surface corresponds to the lowest decreasing rate of the residual pullout resistance. Therefore, the hook angle at the intersection of the pullout stress surface and the 0.5 *f*_u_ surface is the optimal hook angle for different conditions. It can be seen from Figure 7a,b that as the tensile strength and diameter of the steel fibers increased, the residual pullout resistance of the steel fibers decreased faster, resulting in a smaller optimal hook angle.

## 4. Steel Fiber Pulling out Behavior Test

To verify whether the optimized steel fibers had better pulling out behavior, different types of hooked end steel fibers were tested, and the pullout resistance—displacement curve was obtained. The pull out behavior of steel fibers after optimization was compared with that of the steel fibers before optimization, and the effects of the shape, diameter, and tensile strength of the steel fibers on the pullout resistance were analyzed.

### 4.1. Specimen Preparation and Testing

**Figure 8:**
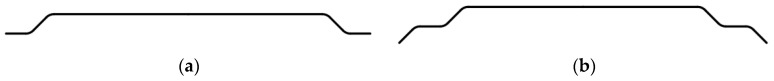
The shape of hooked-end steel fiber. (**a**) 135-2/146-2 hooked-end steel fiber; (**b**) 135-3 hooked-end steel fiber.

P-O 42.5 ordinary silicate cement and ordinary river sand were used for the test. The parameters of the hooked end steel fibers are shown in Table 2, and their shapes are shown in Figure 8. The mixing ratios of the cementitious composites were water:cement:sand = 0.42:1:2 and water:cement:sand = 0.50:1:1.7. The 146-2-1100-0.75 hooked end steel fiber was the result of optimization of the steel fiber with a diameter of 0.75 mm and tensile strength of 1100 MPa under a matrix with a water-cement ratio of 0.50.

**Figure 9:**
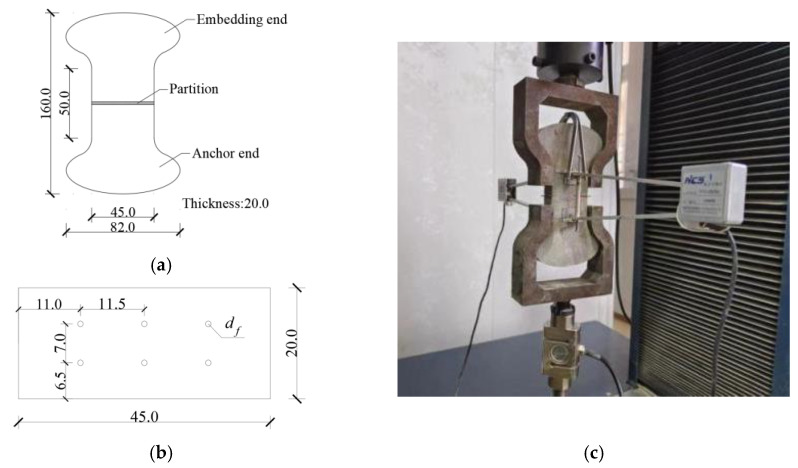
The test method of pullout resistance of steel fiber. (**a**) Specimen size (mm); (**b**) Partition size (mm); (**c**) The device diagram steel fiber pull-out test.

The test adopted the “dog-bone” specimen, which was divided into the embedded end and anchored end. The ends were separated using a metal partition. The specimen size is shown in Figure 9a, and the partition size is shown in Figure 9b. The anchored end of the specimen was first poured followed by an embedded section after 24 h. After removing the mold, the specimen was placed in a curing chamber under standard conditions for 28 days. Six fibers were used in the specimens, and three specimens were prepared for each group.

The steel fiber pulling out behavior test was carried out on a universal testing machine according to the test method in the Standard of Test Methods for Steel Fiber Concrete (CECS13:89) [21], with displacement control throughout the test and a loading rate of 0.4 mm/s, as shown in Figure 9c.

### 4.2. Test Results

**Figure 10:**
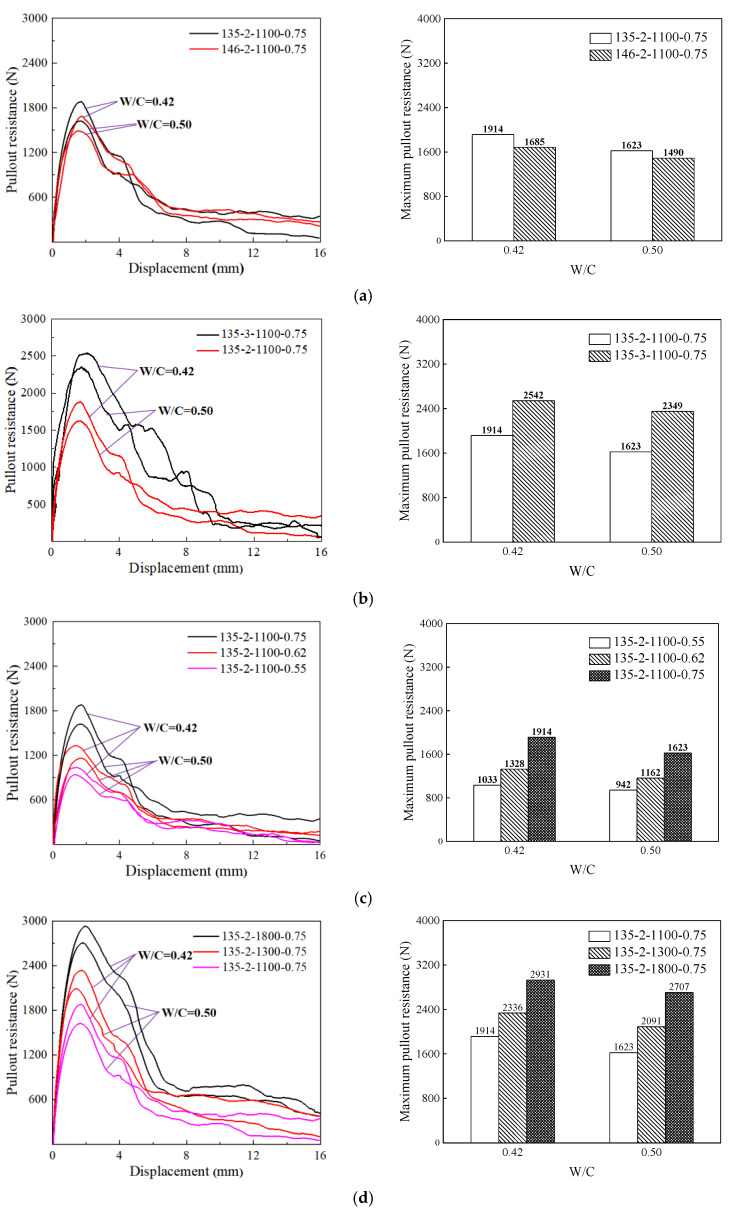
The pullout resistance-displacement curves and the maximum pullout resistance of different types of hooked-end steel fibers. (**a**) The pullout resistance-displacement curves and the maximum pullout resistance of steel fiber with different hook angles; (**b**) The pullout resistance-displacement curves and the maximum pullout resistance of steel fiber with different hook numbers; (**c**) The pullout resistance-displacement curves and the maximum pullout resistance of steel fibers with different diameters; (**d**) The pullout resistance-displacement curves and the maximum pullout resistance of steel fiber with different tensile strength.

The pullout resistance-displacement curves are shown in Figure 10.

**Figure 11:**
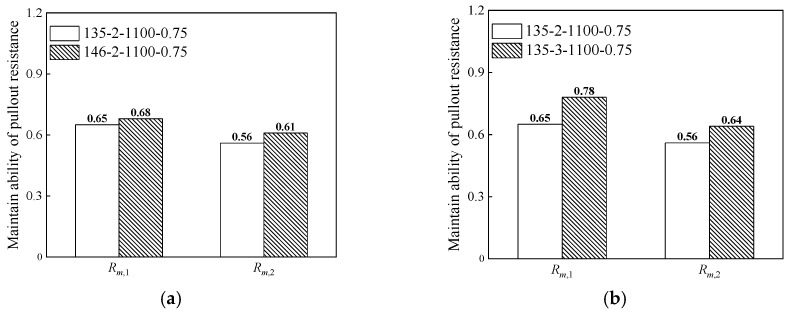
The effect of steel fiber shape on the ability to maintain pullout resistance. (**a**) The influence of hook angle on the ability to maintain pullout resistance; (**b**) The influence of the number of hooks on the ability to maintain pullout resistance.

The effects of the hook angle, the number of hooks, diameter and tensile strength on the maximum pullout resistance are shown in Figure 10 and Figure 11, respectively. As shown in Figure 11a, the maximum pullout resistance of the steel fibers increased by 14% (W/C = 0.42) and 9% (W/C = 0.50) when the hook angle is adjusted from 146° (146-2-1100-0.75) to 135° (135-2-1100-0.75). As shown in Figure 11b, the maximum pullout resistance of the steel fibers increases by 33% (W/C = 0.42) and 45% (W/C = 0.50) when the number of hooks increased from 2 (135-2-1100-0.75) to 3 (135-3-1100-0.75). Similarly, when the diameter of the steel fiber increases from 0.55 mm (135-2-1100-0.55) to 0.75 mm (135-2-1100-0.75), the maximum pullout resistance of the steel fiber increases by 85% (W/C = 0.42) and 72% (W/C = 0.50). The maximum pullout resistance of the steel fibers increased by 53% (W/C = 0.42) and 67% (W/C = 0.50) when the tensile strength of the steel fiber increased from 1100 MPa (135-2-1100-0.75) to 1800 MPa (135-2-1800-0.75). In summary, with a decreasing hook angle, increasing number of hooks, increasing diameter, and increasing tensile strength, the maximum pullout resistance of the steel fiber increased. In addition, the pullout stress (562 MPa) of the optimized hooked end steel fiber was greater than 0.5 *f_u_* (550 Mpa).

It can be seen from Figure 10 that the greater the stiffness, the greater the maximum pullout resistance. However, the greater the stiffness, the worse the ductility of the steel fiber. Therefore, the fibers are more prone to fracture. The smaller the hook angle of the steel fiber and the greater the number of hooks, the better the toughness of the steel fiber.

The ability to maintain the pullout resistance of steel fibers has an important effect on pull out behavior. In this study, the ratios of the residual pullout resistances *P*_1_ and *P*_2_ to the maximum pullout resistance *P_m_* at pullout displacements of 3 and 4 mm were used to evaluate the ability of the steel fibers to maintain pullout resistance (*R_m_*_,1_ and *R_m_*_,2_). A larger ratio of hooked-end steel fibers leads to a stronger ability to maintain the pullout resistance and better pulling out behavior. However, a smaller ratio of hooked end steel fibers results in a weaker ability to maintain pullout resistance and worse pulling out behavior.

The effect of steel fiber shape on the ability to maintain pullout resistance is shown in Figure 11. As shown in Figure 11a, when the hook angle was adjusted from 135° (135-2-1100-0.75) to 146° (146-2-1100-0.75, optimized), *R_m_*_,1_ and *R_m_*_,2_ increased by 5 and 9% (W/C = 0.50), respectively. This result indicates that the ability of the steel fibers to maintain pullout resistance improves as the bending hook angle decreases. After optimization, the pullout resistance of the steel fibers decreased more slowly. As shown in Figure 11b, when the number of hooks increased from 2 (135-2-1100-0.75) to 3 (135-3-1100-0.75), *R_m_*_,1_ and *R_m_*_,2_ increased by 20% and 14% (W/C = 0.50), respectively.

## 5. Analysis and Discussion

The geometric L/D ratio of steel fibers (i.e., the ratio of length—to—diameter) is an important parameter of steel fibers and is commonly used to characterize its reinforcing effect [22]. The pullout resistance of straight steel fibers is only related to the interfacial shear stress and surface area of the steel fibers. Therefore, the L/D ratio can objectively characterize the pull out behavior of straight steel fibers in the same matrix. However, hooked end steel fibers have a mechanical anchoring effect, resulting in a significantly larger pullout resistance than straight steel fibers. Therefore, the geometric L/D ratio cannot accurately reflect the reinforcing effect of hooked end steel fibers, and more reasonable steel fiber parameters should be employed to analyze SFRCC. On this basis, an equivalent L/D ratio was proposed. Steel fibers of any shape were equivalent to straight steel fibers according to the same maximum pullout resistance, and the geometric L/D ratio of the equivalent straight steel fibers was used as the equivalent L/D ratio. Due to the anchoring effect of the hooks, the equivalent L/D ratio of the hooked-end steel fibers was greater than the geometric L/D ratio, whereas that of the straight steel fibers was equal to the geometric L/D ratio.

When the water-cement ratio of the cementitious matrix was 0.50, the maximum resistance when pulling out a single 135-2-1100-0.75 hooked end steel fiber was 270 N. If the steel fibers are in a straight shape, the bonding shear stress between the steel fiber and the matrix is 2.2 MPa. At the same pullout force, the length was 104 mm, and the equivalent L/D ratio of the 135-2-1100-0.75 hooked end steel fiber was 139. The equivalent L/D ratios of the remaining hooked end steel fibers in this cementitious matrix is shown in Table 3.

To verify the superiority of the equivalent L/D ratio over the geometric L/D ratio in characterizing the reinforcing effect of steel fibers, the flexural properties of steel fiber concrete analyzed using both L/D ratios were compared with the flexural properties measured by the tests. The flexural properties of 135-2-1800-0.75 type hooked end steel fiber—reinforced concrete were tested by using the material mix ratio shown in Table 4, and the specimens were prepared and tested according to the European standard EN 14651 [23]. The residual flexural-tensile strength *f_R_*_,1_, *f_R_*_,2_, *f_R_*_,3_, and *f_R_*_,4_ of hooked-end steel fiber concrete notched beams were measured to be 3.4 MPa, 3.4 MPa, 2.7 MPa, and 2.4 MPa, respectively.

Ding et al. [19] proposed a calculation method for the residual flexural-tensile strength, as expressed in Equation (8).

(8) $$f_{R,i}=\left\{\begin{array}{c} 0.52f_{t}+2.82V_{f}\lambda \tau_{s}+0.0026V_{f}f_{y}\;\;\;\;i=1\\ 0.38f_{t}+3.04V_{f}\lambda \tau_{s}+0.0094V_{f}f_{y}\;\;\;\;i=2\\ 0.22f_{t}+3.16V_{f}\lambda \tau_{s}+0.1046V_{f}f_{y}\;\;\;\;i=3\\ 0.15f_{t}+2.96V_{f}\lambda \tau_{s}+0.1072V_{f}f_{y}\;\;\;\;i=4 \end{array}\right.$$

where *f_t_* is the matrix cracking strength, which was 4.8 MPa in this study, *V_f_* is the steel fiber dosage, λ is the geometric L/D ratio of the steel fiber, $\lambda =l_{f}/d_{f}$; *l_f_* is the length of the steel fiber; *τ_s_* is the interface shear stress between the steel fiber and the matrix.

The residual tensile strengths were calculated by replacing the geometric L/D ratio in Equation (8) with the equivalent L/D ratio. The residual flexural-tensile strength calculated from the geometric L/D ratio was small, and that calculated from the equivalent L/D ratio was closer to the test value. Therefore, the equivalent L/D ratio can more accurately reflect the reinforcing effect of the steel fibers.

## 6. Conclusions

The shape of the hooked end steel fiber was optimized by considering factors such as the hook angle, number of hooks, diameter, and tensile strength. The pull out behavior of the different hooked end steel fibers was evaluated using a pullout resistance test. The main conclusions are as follows:

(1) An optimization method for the steel fiber shape was proposed to change the hook angle and number of hooks to slow down the decrease in pullout resistance under a certain diameter and tensile strength.
(2) The maximum pullout resistance of the hooked end steel fibers increased with decreasing hook angle, increasing number of hooks, increasing diameter, increasing tensile strength, and increasing matrix strength.
(3) Optimized steel fibers have a stronger ability to maintain pullout resistance than preoptimized steel fibers. When the water-cement ratio of the cementitious matrix is 0.5, and the steel fiber diameter is 0.75 mm with a tensile strength of 1100 MPa, the ability of the steel fiber to maintain pullout resistance (*R_m_*_,1_ and *R_m_*_,2_) increases by 5% and 9% after adjusting the hook angle from 135° to 146° (optimized), respectively.
(4) Compared with the residual flexural-tensile strength of steel-fiber concrete notched beams calculated using the geometric L/D ratio, which is calculated using the equivalent L/D ratio is closer to the test value, indicating that the equivalent L/D ratio can more accurately reflect the reinforcing effect of steel fibers.

## Figures and Tables

**Table 1:** The comparison of steel fiber pullout performance before and after optimization.

	Hook Angle (°)	Number of Hooks	Maximum Pullout Resistance (N)	Pullout Stress (MPa)	0.5 *f_u_* *(MPa)*	Residual Pullout Resistance (N)	Pullout Resistance Decline Rate *k*
Before optimized steel fiber a	135	2	259	587	550	143	0.55
Before optimized steel fiber b	160	2	228	516	550	138	0.61
After optimized steel fiber c	146	2	244	553	550	141	0.58

**Table 2:** Parameters of steel fiber.

Steel Fiber Types	Hook Angle (°)	Number of Hooks	Diameter (mm)	Length (mm)	Tensile Strength (MPa)
135-2-1100-0.55	135	2	0.55	60	1100
135-2-1100-0.62	135	2	0.62	60	1100
135-2-1100-0.75	135	2	0.75	60	1100
135-2-1300-0.75	135	2	0.75	60	1300
135-2-1800-0.75	135	2	0.75	60	1800
146-2-1100-0.75	146	2	0.75	60	1100
135-3-1100-0.75	135	3	0.75	60	1100

Note: fiber type 135-2-1100-0.75 represents hook angle-hook number-tensile strength-diameter, respectively.

**Table 3:** The equivalent L/D ratio of the hooked-end steel fibers.

W/C	Steel Fiber Types	Maximum Pullout Resistance (Single) (N)	Geometric L/D Ratio	Equivalent L/D Ratio
0.50	135-2-1100-0.55	157	109	150
	135-2-1100-0.62	194	97	146
	135-2-1100-0.75	271	80	139
	135-2-1300-0.75	349	80	180
	135-2-1800-0.75	451	80	232
	146-2-1100-0.75	248	80	128
	135-3-1100-0.75	392	80	202

**Table 4:** Mix proportion of steel fiber reinforced concrete.

W/C	Water (kg/m^3^)	Cement (kg/m^3^)	Sand (kg/m^3^)	Pebble (kg/m^3^)	Steel Fiber Volume Fraction (kg/m^3^)
0.50	175	350	890	965	20

## Data Availability

Data will be made available on request.
